# Target-specific delivery of doxorubicin to retinoblastoma using epithelial cell adhesion molecule aptamer

**Published:** 2012-11-22

**Authors:** Nithya Subramanian, Vaishnavi Raghunathan, Jagat R. Kanwar, Rupinder K. Kanwar, Sailaja V. Elchuri, Vikas Khetan, Subramanian Krishnakumar

**Affiliations:** 1 Larsen & Toubro Department of Ocular Pathology, Vision Research Foundation, Sankara Nethralaya, Chennai, India; 2 Nanomedicine Laboratory of Immunology and Molecular Biomedical Research (LIMBR), Centre for Biotechnology and Interdisciplinary Biosciences (BioDeakin), Institute for Technology and Research Innovation (ITRI), Geelong Technology Precinct (GTP), Deakin University, Geelong, Victoria, Australia; 3 Nanobiotechnology Laboratory, Vision Research Foundation, Sankara Nethralaya, Chennai, India; 4 Departments of Ocular Oncology and Retina and Vitreous, Medical Research Foundation, Sankara Nethralaya, Chennai, India

## Abstract

**Purpose:**

To study target-specific delivery of doxorubicin (Dox) using an RNA aptamer against epithelial cell adhesion molecule (EpCAM) in retinoblastoma (RB) cells.

**Methods:**

The binding affinity of the EpCAM aptamer to RB primary tumor cells, Y79 and WERI-Rb1 cells, and Müller glial cell lines were evaluated with flow cytometry. Formation of physical conjugates of aptamer and Dox was monitored with spectrofluorimetry. Cellular uptake of aptamer-Dox conjugates was monitored through fluorescent microscopy. Drug efficacy was monitored with cell proliferation assay.

**Results:**

The EpCAM aptamer (EpDT3) but not the scrambled aptamer (Scr-EpDT3) bound to RB tumor cells, the Y79 and WERI-Rb1 cells. However, the EpCAM aptamer and the scrambled aptamer did not bind to the noncancerous Müller glial cells. The chimeric EpCAM aptamer Dox conjugate (EpDT3-Dox) and the scrambled aptamer Dox conjugate (Scr-EpDT3-Dox) were synthesized and tested on the Y79, WERI-Rb1, and Müller glial cells. The targeted uptake of the EpDT3-Dox aptamer caused cytotoxicity in the Y79 and WERI-Rb1 cells but not in the Müller glial cells. There was no significant binding or consequent cytotoxicity by the Scr-EpDT3-Dox in either cell line. The EpCAM aptamer alone did not cause cytotoxicity in either cell line.

**Conclusions:**

The results show that the EpCAM aptamer-Dox conjugate can selectively deliver the drug to the RB cells there by inhibiting cellular proliferation and not to the noncancerous Müller glial cells. As EpCAM is a cancer stem cell marker, this aptamer-based targeted drug delivery will prevent the undesired effects of non-specific drug activity and will kill cancer stem cells precisely in RB.

## Introduction

Retinoblastoma (RB) is the most common intraocular malignant tumor arising in the retina of children, typically diagnosed at 2–3 years of age [1]. Though various treatment strategies are available, in the case of advanced unilateral or bilateral RB, enucleation is the ultimate choice [2]. Thus, newer therapeutic strategies are needed for managing this childhood tumor. Advances in molecular biology have revolutionized treatment strategies for cancer. One such strategy is the development of aptamers [3]. Aptamers are functional nucleic acid ligands that are single-stranded oligonucleotides with specific binding toward the target antigen. Aptamers could be DNA aptamers, RNA aptamers, or peptide aptamers based on the library used for screening. They are generated by a molecular selection process called Systematic Evolution of Ligands by Exponential Enrichment (SELEX) [4].

Aptamers are smaller, are easy to synthesize, and have greater affinity coupled with excellent target specificity and better stability avoiding immunogenicity [3-5]. Aptamers can be functional blocking aptamers such as nucleolin aptamers or target-binding aptamers such as the prostate-specific membrane antigen (PSMA) aptamer for prostate cancer and Mucin 1, the cell surface associated (MUC1) aptamer for breast cancer [6]. Aptamer chimerization has been performed to diversify their use in targeted therapy. The aptamer chimerization process aims to combine two aptamers or an aptamer with another non-aptamer moiety such as biomacromolecules, drugs, or dyes. One combining partner recognizes the target, and the other affects the target molecule’s function [7,8]. Chimerization either through natural recombination or chemical engineering may result in diminishing the activity of one or both recombining partners. Therefore, research investigations are required to study chimeric aptamers [6,9-16].

Cancer cells have different cell types among which exist a subset of cells, with features of stem cells, and are recognized as cancer stem cells (CSC)s or cancer progenitor cells (CPC)s. According to the CSC hypothesis, this subset of cells, having characteristics such as extensive proliferation, self-renewal, and differentiation to multiple lineages, thus act as tumor-initiating cells [17]. Their existence has opened up a new avenue of drug targeting. Progenitor cells have these features, and it could be hypothesized that the CSCs may arise from mutation of such progenitor cells, which usually lack the self-renewal characteristic [18]. There is no clear evidence of the origin of cancer stem cells, and in the case of the breast tissue differentiation model, epithelial cell adhesion molecule (EpCAM) acts more like a progenitor cell than a stem cell [19]. Similarly, in the case of hepatocellular carcinoma, EpCAM^+^ α-fetoprotein^+^ cells show characteristics of CSCs/CPCs [20].

Cancer stem cells for several malignancies are capable of unlimited self-renewal and differentiation leading to tumorigenicity, cancer recurrence, and metastasis [21-24]. These cells are chemotherapy and radiation therapy resistant. Therefore, targeting these cells with newer therapeutic agents will eradicate the relapse and metastasis. EpCAM is a putative cancer stem cell marker and is dysregulated in several epithelial cancers [25-29]. Earlier, we showed that EpCAM is overexpressed in RB tumors, with choroid or optic nerve invasion [30]. Therefore, EpCAM is an ideal target molecule for RB therapy. EpCAM gene silencing using small interfering RNA (siRNA) reduced RB cell proliferation [31]. Cancer immunotherapy by using a bispecific EpCAMXCD3 antibody to redirect the T lymphocytes to target the EpCAM-positive CSCs reduced cell proliferation [32]. Nanocarriers’ functionalized EpCAM antibody delivered the anticancer drug paclitaxel to target EpCAM-positive CSCs in RB [33]. Various other immunotherapy-based clinical trials on pancreatic, ovarian, and gastric cancers using anti-EpCAM antibodies are in progress [34].

Recently, an RNA aptamer was isolated against the cancer stem cell marker EpCAM, by cell surface SELEX for proposed theranostic applications in EpCAM-positive cancer cells [35]. Chimeric EpCAM aptamer functionalized with groups such as locked nucleic acid using supraparamagnetic iron oxide nanoparticles showed efficacy in killing cancer cells [6]. However, studies are lacking on the use of other molecules with conjugated EpCAM aptamer to target the stem cell marker, EpCAM.

Doxorubicin (Dox) is a Food and Drug Administration–approved drug commonly used to treat some leukemia and Hodgkin's lymphoma, as well as cancers of the bladder, breast, stomach, lung, ovaries, thyroid, soft tissue sarcoma, multiple myeloma, and RB [36]. The molecular mechanism behind the cellular toxicity created by Dox is by intercalation with the nucleic acids and inhibiting them in further functional activities [37]. We used this property of Dox for the study, by intercalating it to EpDT3 to deliver it to EpCAM-expressing cancer stem cells. Previously, Dox-conjugated PSMA aptamer or scgc8 aptamers were shown to cause cell-specific cytotoxicity [12,38]. Recently, use of sonoporation for the enhanced delivery of Dox using microbubbles in RB cells was reported [39]. Therefore, specific targeting of CSCs using carrier systems will improve drug efficacy to treat various cancers. Hence, in the current study we created an EpDT3-Dox conjugate to target cancer stem cells using the RB cell line as a model. The results indicated that the aptamer-Dox conjugate can specifically target cancer stem cells compared to noncancerous Müller glial cells.

## Methods

### Cell culture

The RB cell lines (Y79 and WERI-Rb1) endogenously expressing EpCAM were obtained from the cell bank, RIKEN BioResource Center (Ibaraki, Japan) and were cultured in RPMI-1640 media. A noncancerous Müller glial cell line derived from the neural retina was a gift from Dr. G.A. Limb (UCL Institute of Ophthalmology, London, England) and was cultured in Dulbecco’s modification of Eagle’s media (DMEM). RPMI-1640 and DMEM were purchased from Sigma Aldrich (Bangalore, India). Fetal bovine serum (FBS) was purchased from Gibco-BRL (Rockville, MD). The RB cell lines were cultured in RPMI-1640 medium, supplemented with 10% FBS and 1X penicillin-streptomycin antibiotics (Himedia) at 37 °C in a 5% CO_2_ humidified incubator. Fresh RB tumor samples were obtained after informed consent was received from the patients. The study adhered to the tenets of the Declaration of Helsinki. This study was approved by the Vision Research Foundation ethics boards and was conducted at the Vision Research Foundation, Sankara Nethralaya, India.

### RNA aptamers

EpCAM aptamer (EpDT3) and scrambled aptamer (Scr-EpDT3) with and without fluorescein (FI) fluorophore were custom synthesized by Dharmacon Inc. (Lafayette, CO). The sequence of the aptamer is 5′-GCG ACU GGU UAC CCG GUC G-3′ [31]. Both aptamers have identical sequences except 2’ modifications. EpDT3 has 2’-Fluoro modified pyrimidines, and Scr-EpDT3 has 2’-OMe modified pyrimidines. Methyl modification at the 2’ position hampers binding of the aptamer to the receptor [31]. Therefore, the aptamer served as negative control to study the functional role of the aptamers. A fluorescent (FI) labeled aptamer was used for the flow cytometry studies. Aptamers were annealed at 70 °C before the conjugation or to the binding experiments.

### Analysis of epithelial cell adhesion molecule expression with flow cytometry

For studying EpCAM expression, cells were washed two times with 1X phosphate buffered saline (PBS; 1X PBS, prepared from 10X PBS, Himedia, Mumbai, India) and resuspended in blocking buffer (PBS with 0.02% sodium azide and 0.1% BSA [BSA]). The cells were incubated with the anti-EpCAM C-10 primary antibody (Santa Cruz Biotechnology, Santa Cruz, CA) at 4 °C for 1 h, washed twice with PBS, followed by incubation with the fluorescein isothiocyanate conjugated anti-mouse immunoglobulin G secondary antibody (Sigma Aldrich) in blocking buffer for 45 min in the dark, and then followed by two washes with PBS. The fluorescence signal was read using flow cytometry (FACS Calibur flow cytometer; BD Biosciences, San Jose, CA), using the CellQuest software program (BD Biosciences).

### Cell-surface binding of aptamer

The specific binding of the EpDT3 aptamer to the fresh tumors and RB cell lines was determined with fluorescein-labeled aptamers. The RB tumor cells (homogenized and suspended in PBS; n=4), the Y79 and WERI-RB1 cells, were washed twice with PBS (1X). The Müller glial cells were washed in PBS with 0.53 mM EDTA followed by two washes with 1X PBS. About 100 nM of FI-labeled RNA aptamers (EpDT3-FI and Scr-EpDT3-FI) were added to 2×10^5^ cells resuspended in 100 µl binding buffer (PBS containing 5 mM MgCl_2_, 0.1 mg⁄ml tRNA, and 0.1 mg⁄ml salmon sperm DNA). The cells were incubated on ice for 1 h followed by three washes in 1X PBS [31]. The cells were stained with propidium iodide (Sigma Aldrich; 1: 20,000) for 5 min, and the signal was read with the flow cytometer [33]. The fluorescence excitation and emission were 488 nm and 530 nm, respectively.

### Aptamer-doxorubicin conjugation

Dox (fluorescent grade, Sigma Aldrich, Bangalore, India) was conjugated to EpDT3 and Scr-EpDT3 in conjugation buffer (0.1 M sodium acetate, 0.05 M NaCl, and 0.1 M MgCl_2_). Aptamer-Dox conjugation was performed by increasing the molar ratios of the aptamer (0, 0.01, 0.1, 1, 3, 5, and 7 equivalents) to constant Dox (3 µM). Fluorescence quenching of the Dox due to the intercalation of Dox to the aptamer (aptamer conjugation) was monitored using spectrofluorimetry (SpectraMax M2 spectrofluorophotometer; Molecular Devices, Sunnyvale, CA) at a constant excitation at 470 nm [33]. The Dox-conjugated aptamers (1:1 molar ratio) were purified from the free Dox by passing through a NAP 10 column (G25-DNA grade column, GE Healthcare, Bangalore, India).

### Release and diffusion of doxorubicin from the aptamer-doxorubicin conjugates

Drug release and diffusion from the chimeric aptamer in vitro were studied by monitoring the passage of Dox under conditions that simulate the physiologic conditions [34]. About 100 µl of the aptamer-Dox conjugates were dialyzed (3.5 kDa cut-off, Pierce) in conjugation buffer at 37 °C. Samples were collected at various time intervals (2 h, 4 h, and 6 h) and were monitored with UV-VIS spectroscopy (Molecular Devices). Free Dox (50 μM) dialyzed in a similar way served as the control. The diffused Dox from the conjugate was measured at 470 nm excitation and 585 nm emission.

### Visualization of aptamer-doxorubicin uptake with fluorescent microscopy

The Y79 cells and the Müller glial cells (2×10^4^ cells/ml) were grown on coverslips coated with poly-L-Lysine. Cells were washed twice with media (without FBS) and treated with equal concentrations (1.5 µM) of aptamer-Dox and free Dox for 2 h and 12 h. The cells were washed twice with 1X PBS, fixed in 3.7% formaldehyde for 10 min, and stained with DAPI for 15 min. The coverslips with cells were washed with 1X PBS and were analyzed under a fluorescence microscope (Axio Observer, Carl Zeiss, Berlin, Germany).

### Cell proliferation assay

The ability of EpDT3-Dox to reduce stem cell proliferation was tested with 3-(4, 5-dimethylthiazol-2-yl)-2, 5-diphenyltetrazolium bromide (MTT) assay. The Y79 cells and WERI-Rb1 cells expressing EpCAM and the non-expressing Müller glial cells (5×10^3^ cells) were treated with equal concentrations (3 μM) of Dox, EpDT3-Dox, and Scr-EpDT3-Dox conjugates for 2 h in serum-free media. The cells were washed and cultured in fresh media containing 10% FBS for 24 h and 48 h. Cytotoxicity was evaluated with an MTT assay. Briefly, 5 µg/ml MTT solution was added to the cells 37 °C for 4 h. The MTT solution was removed, and 100 μl DMSO was added to dissolve MTT crystals. The assay was read using a microplate reader at 570 nm absorbance.

### Statistical analysis

The statistical significance was calculated using an unpaired Student *t* test. The asterisk represents values less than or equal to 0.05.

## Results

### Epithelial cell adhesion molecule aptamer binds to retinoblastoma tumor cells and cell lines

**Figure 1:**
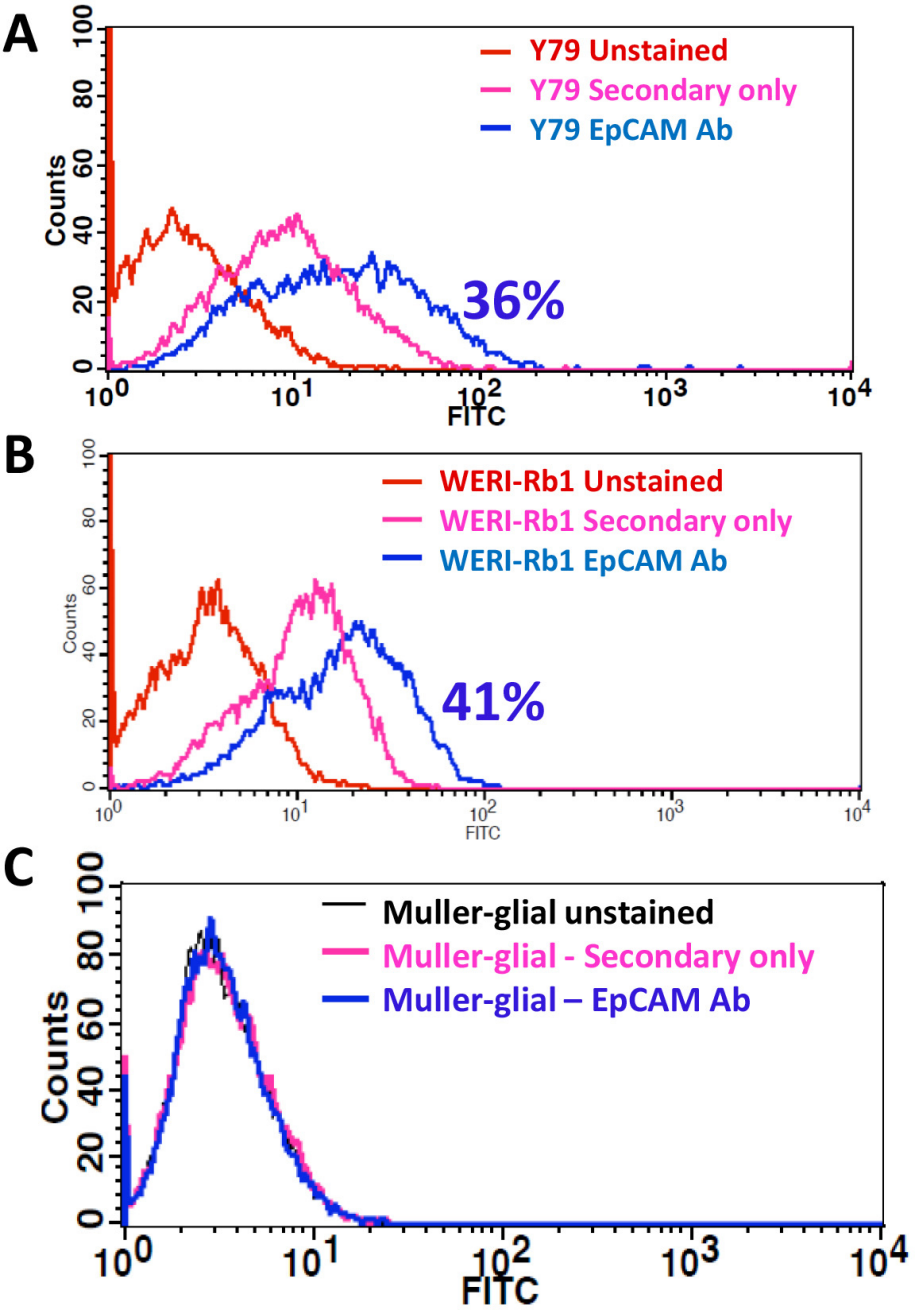
EpCAM expression on cell lines. Y79, WERI-Rb1, and Müller glial cells were stained for EpCAM expression with indirect immunofluorescence followed by flow cytometry. **A**, **B**, **C**: Y79, WERI-Rb1, and Müller glial cells stained either with secondary antibody alone or primary anti-EpCAM (C-10) antibody, followed by secondary anti-mouse fluorescein isothiocyanate antibody and flow cytometry. The overlay plot shows the positive population.

**Figure 2:**
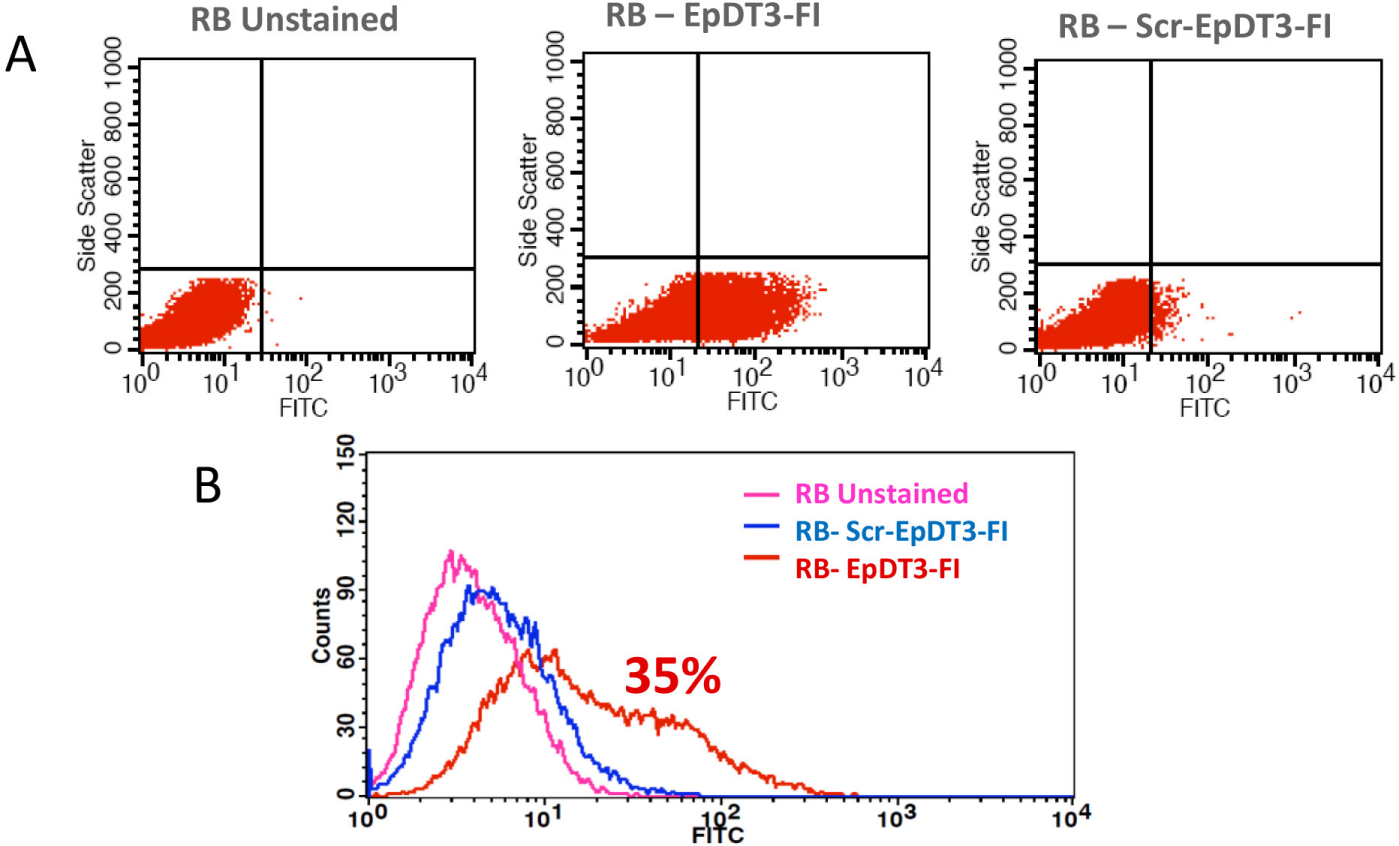
EpCAM aptamer binding on RB primary cells. Fluorescently labeled EpCAM aptamers were incubated with RB cells obtained from fresh tumor samples and analyzed with flow cytometry. Dead cells were gated out by gating the propidium iodide–positive cells. **A**, **B**, **C**: Scatter plots show control unstained RB tumor cells, tumor cells stained with EpDT3-FI shifting toward the right, and Scr-EpDT3-FI showing the least binding. **D**: Histogram overlay plot showing significant binding of EpDT3-FI to tumor cells compared to Scr-EpDT3-FI.

**Figure 3:**
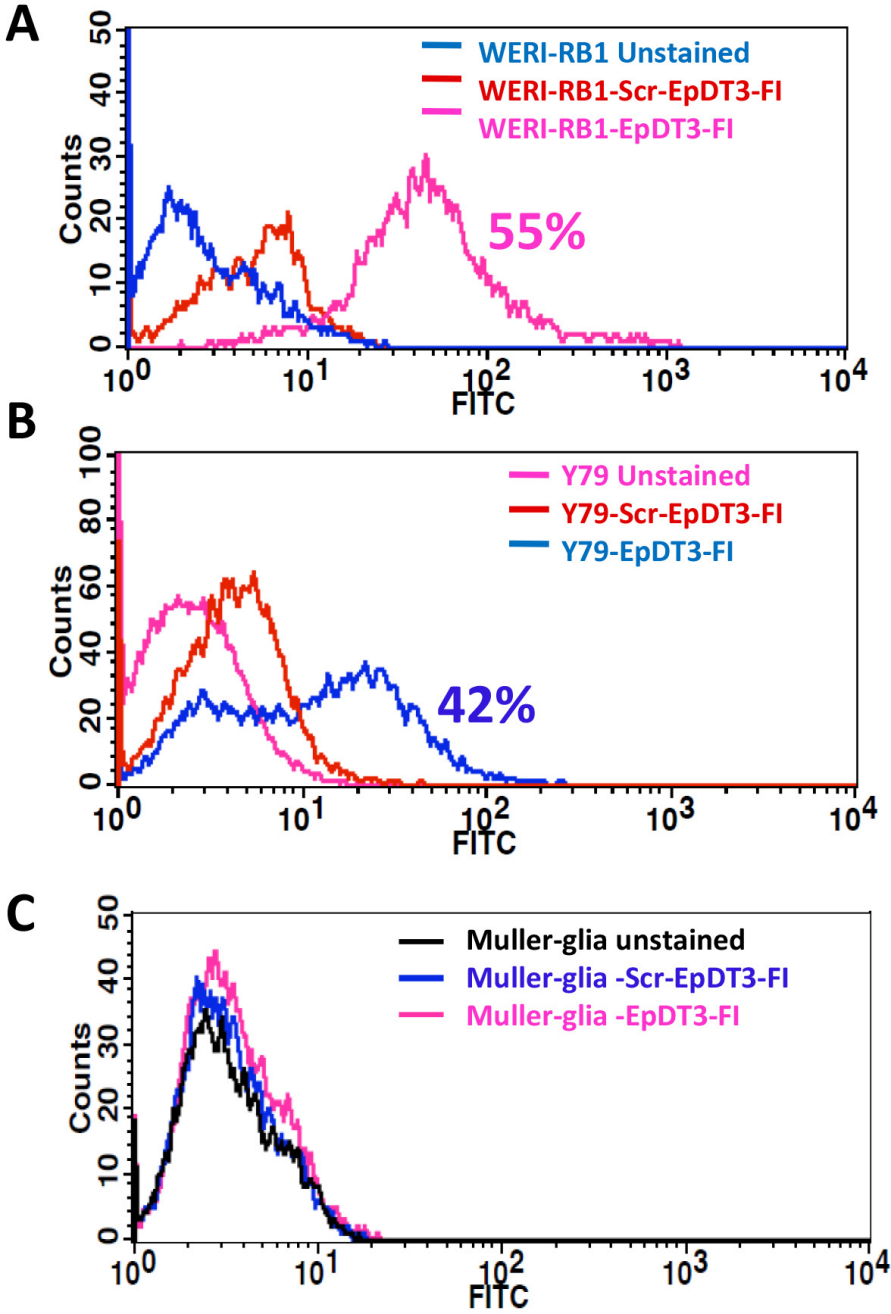
EpCAM aptamer binding on cell lines. Fluorescently labeled EpCAM aptamers were incubated with RB cell lines and Müller glial cells and analyzed with flow cytometry. Histogram overlay plot showing distinct population of (**A**) WERI-Rb1 and (**B**) Y79 cells bound to EpDT3-FI compared to unstained and Scr-EpDT3-FI bound cells. **C**: Histogram overlay plot of Müller glial cells showing no distinct population upon incubation with EpDT3-FI or Scr-EpDT3-FI from unstained cells.

Equal EpCAM expression of around 35%–40% positive cells was observed in the Y79 and WERI-RB1 cell lines, whereas the noncancerous Müller glial cells showed no expression of the protein (Figure 1A-C). EpDT3-FI bound to 35% of the RB tumor cells (n=4; Figure 2A,B), Y79 and WERI-Rb1, whereas Scr-EpD3-F1 did not bind to all the RB tumor cells or cell lines (Figure 3A,B). The Müller glial cells showed no binding for either aptamer (Figure 3C).

### Preparation of aptamer-doxorubicin conjugates

**Figure 4:**
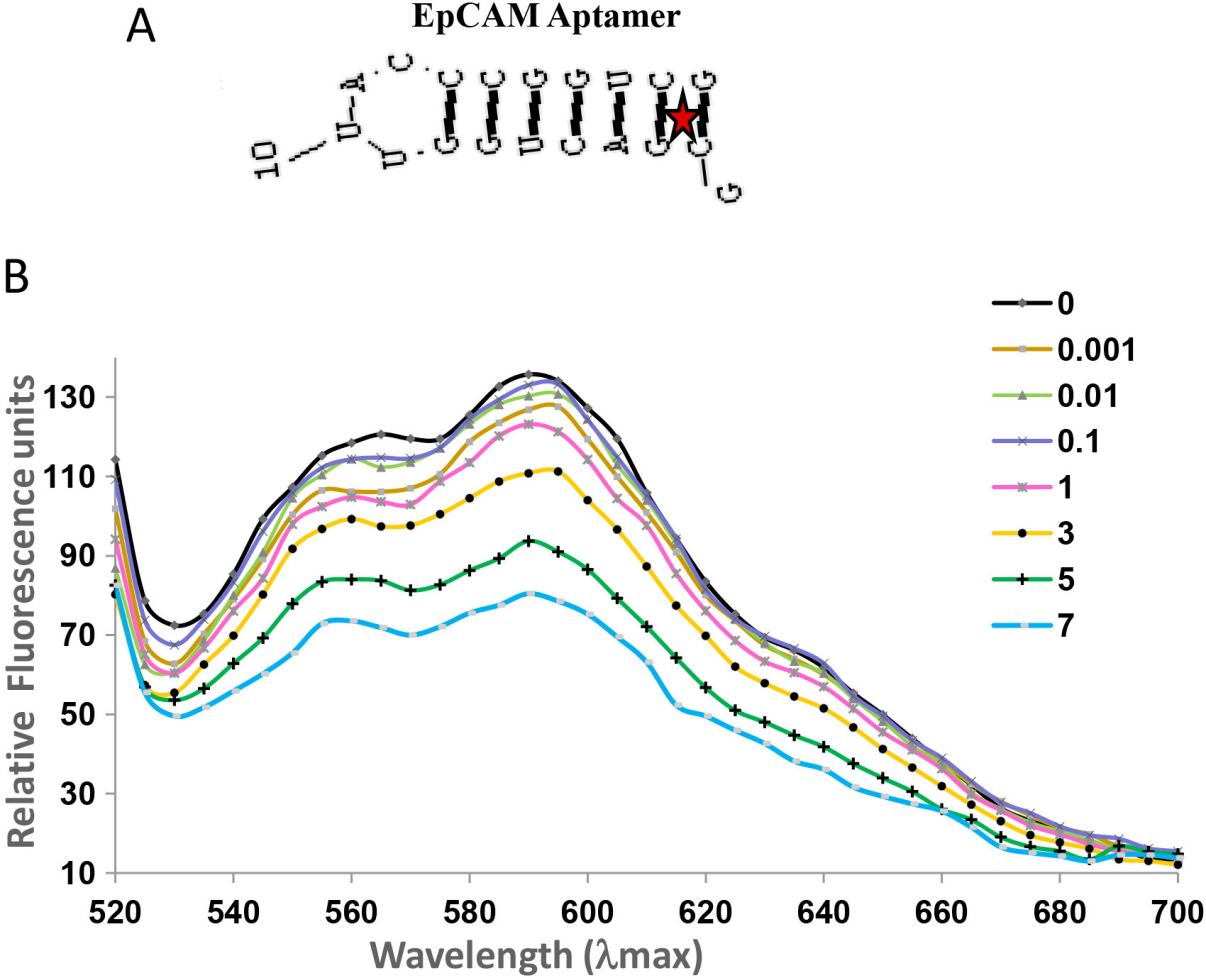
EpCAM aptamer structure and doxorubicin conjugation. Structure prediction was performed using the RNA Structure program. **A**: Hairpin structure of the EpCAM aptamer with the predicted site for the doxorubicin intercalation represented as a red star. **B**: Fluorescence spectra of doxorubicin (3.0 μm) with increasing molar ratios of the aptamer (from top to bottom: 0, 0.01, 0.1, 1, 3, 5, and 7 equivalents) in conjugation buffer.

The site of Dox intercalation in the EpDT3 aptamer, for cancer cell–specific drug delivery was analyzed with RNA structure program version 5.3. The predicted EpDT3 secondary structure consists of a hairpin structure, and the site of Dox intercalation is between the GC and CG sequence in the aptamer and has a single site for Dox intercalation (Figure 4A). Following the prediction, we optimized the aptamer-Dox conjugation assay and observed gradual quenching of fluorescence from Dox as the aptamer concentration increased (Figure 4B). The EpDT3-Dox and Scr-EpDT3-Dox conjugates generated were used for functional studies.

### Release and diffusion of the drug from the aptamer-doxorubicin conjugate

**Figure 5:**
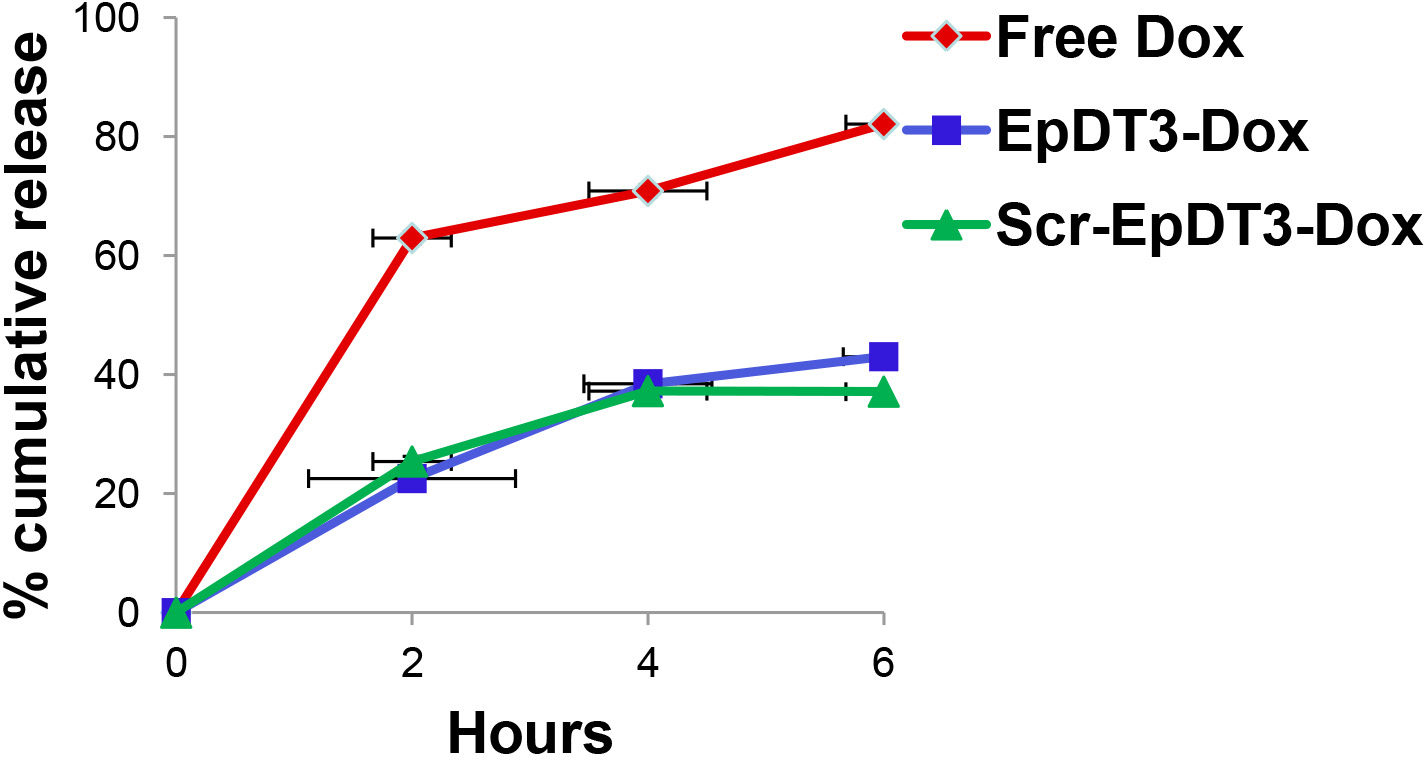
Diffusion of doxorubicin from the aptamer-Dox conjugate. The time-dependent release of doxorubicin from aptamer-Dox conjugates was monitored by dialyzing free Dox and aptamer-Dox conjugates against conjugation buffer. Dox from the aptamer-Dox conjugate (closed square and triangle) and the free Dox (closed diamond) released were monitored from 2 to 6 h of the initiation of dialysis and represented as % cumulative release. Aptamer-Dox conjugates release Dox comparatively slower than free Dox.

The release and diffusion of the drug from the Dox-conjugated aptamer were studied under artificial conditions mimicking the role of the cell membrane (Figure 5). The percent cumulative release of the Dox from the chimeric aptamers was onefold less than the free Dox. The dissociation of Dox from the Dox-conjugated aptamer was about 20%, 37%, and 45% by 2 h, 4 h, and 6 h, respectively. The free Dox dissociated much faster than the aptamer-Dox (60%, 70%, and 80% by 2 h, 4 h, and 6 h, respectively).

### Targeted delivery and uptake of doxorubicin in the cell line

**Figure 6:**
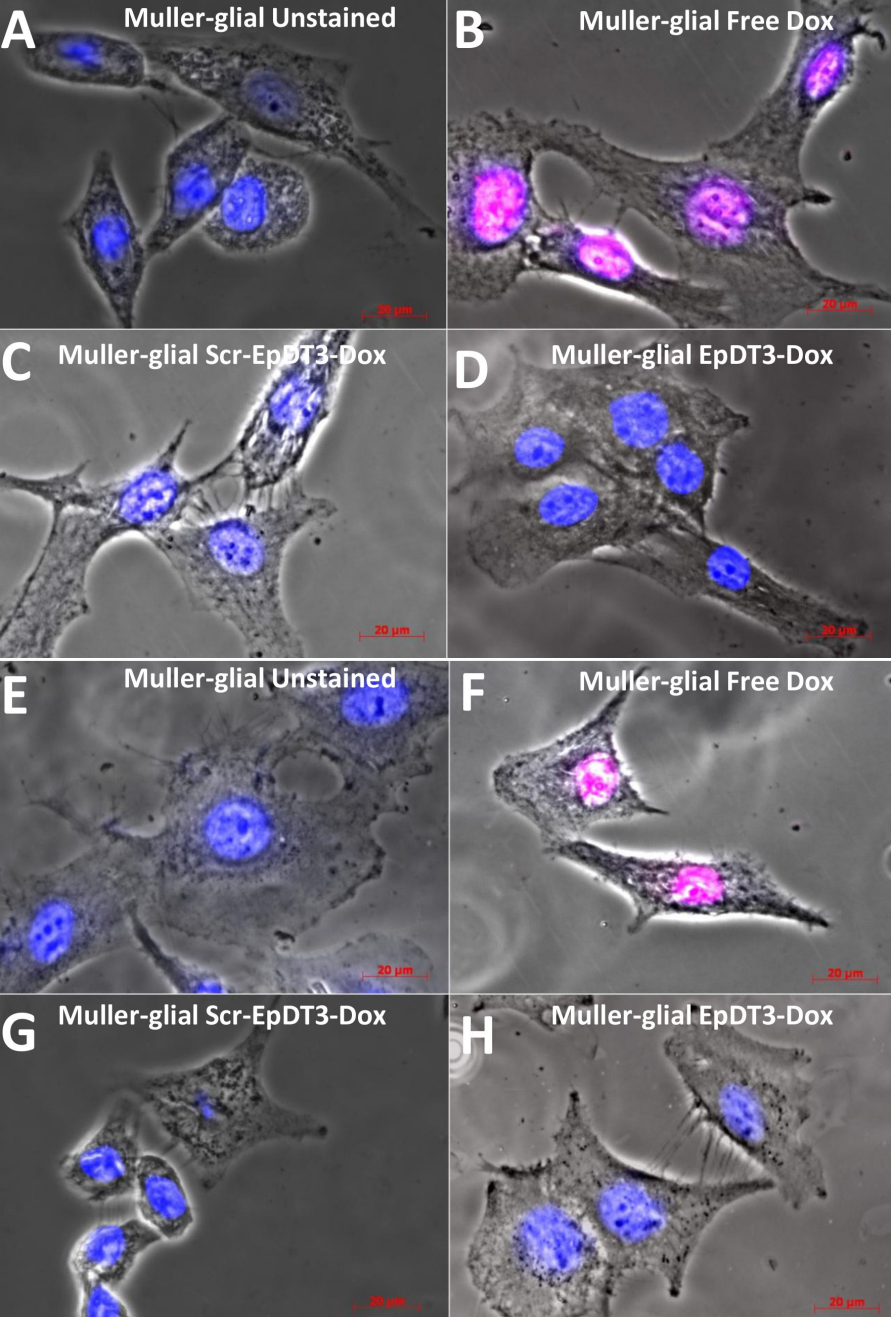
Uptake of aptamer-Dox conjugate on Müller glial cells addition in vitro. Free Dox, EpDT3-Dox, and Scr-EpDT3-Dox were incubated with Müller glial cells for 2 h and observed under an AxioObserver microscope. **A**, **B**, **C,** and **D**: 2 h incubated cells unstained, treated with free Dox, Scr-EpDT3-Dox, or EpDT3-Dox. **E**, **F**, **G**, and **H**: 12 h incubated post 2 h of treatment with free Dox, Scr-EpDT3-Dox, or EpDT3-Dox.

**Figure 7:**
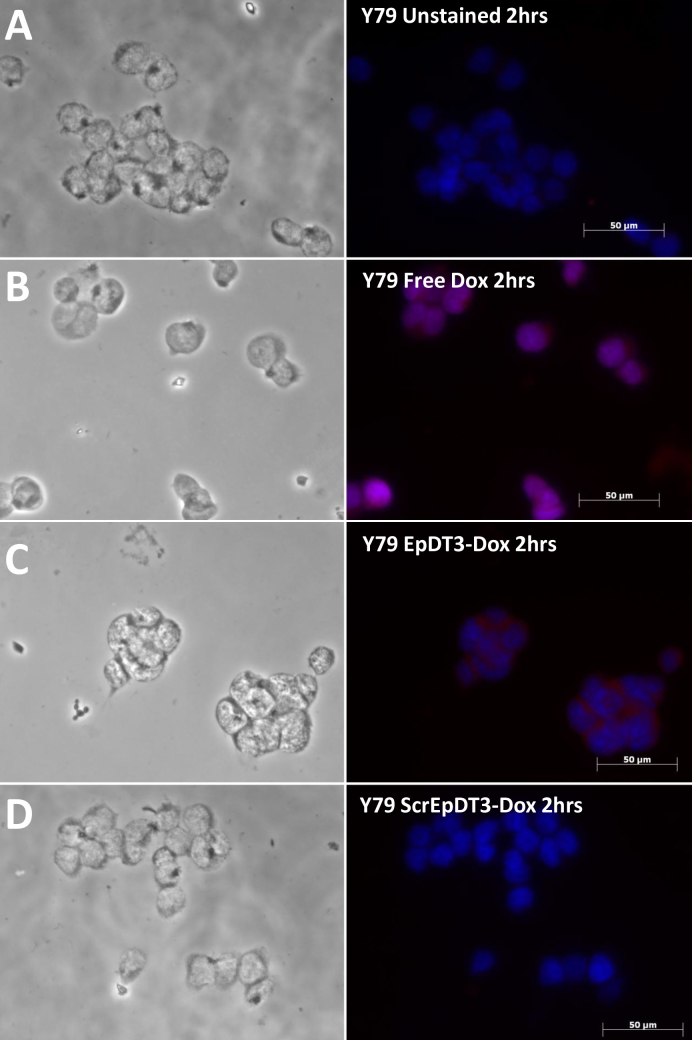
Uptake of aptamer-Dox conjugate on the Y79 cell line post 2 h addition in vitro. Free Dox, EpDT3-Dox, and Scr-EpDT3-Dox were incubated with Y79 cells for 2 h and observed under an AxioObserver microscope. **A**, **B**, **C**, and **D**: phase images of Y79 cells unstained, treated with free Dox, Scr-EpDT3-Dox or Scr-EpDT3-Dox and the respective right panel represents the merged fluorescence image.

**Figure 8:**
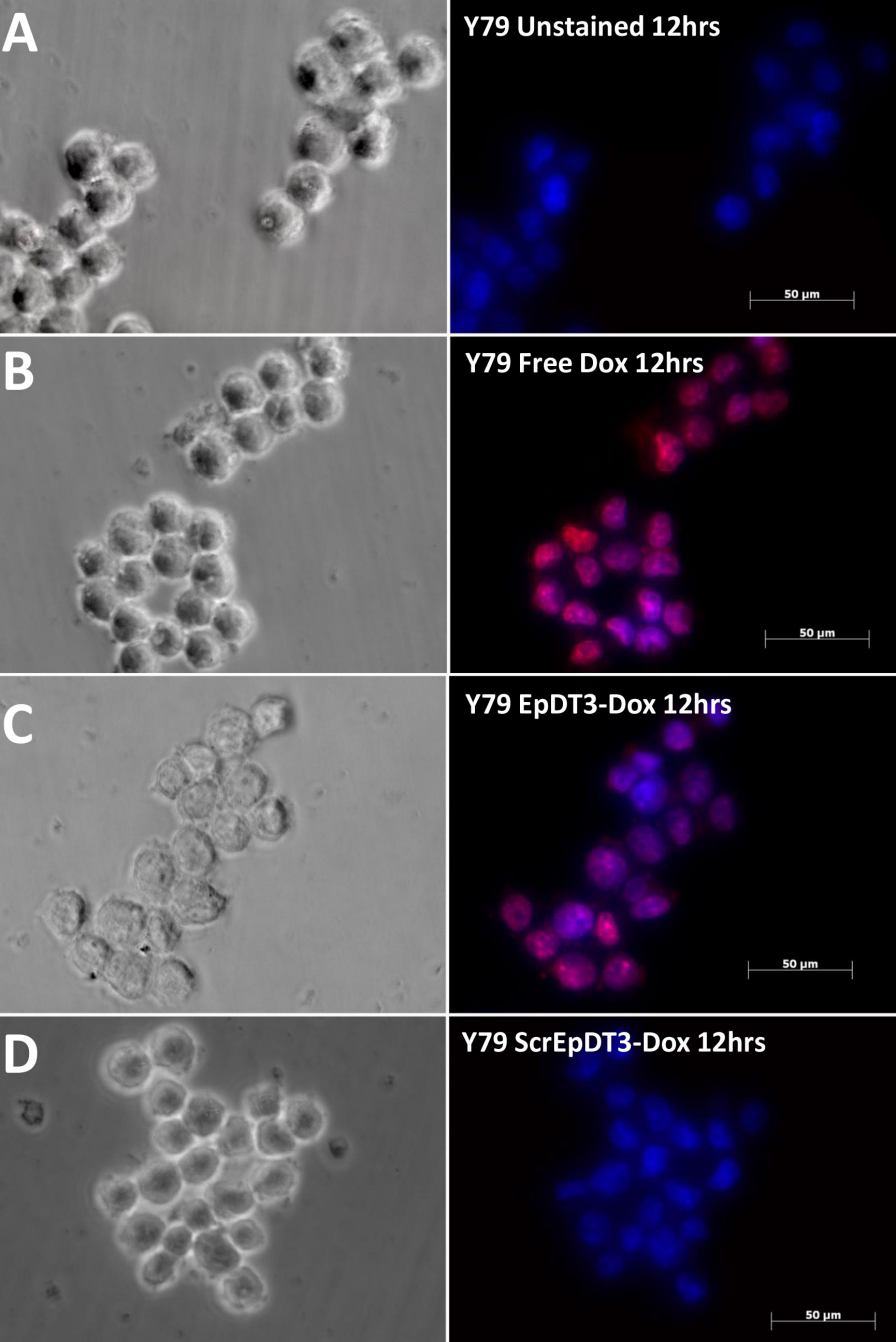
Uptake of aptamer-Dox conjugate on the Y79 cell line post 12 h addition in vitro. Free Dox, EpDT3-Dox, and Scr-EpDT3-Dox were incubated with Y79 cells for 12 h and observed under an AxioObserver microscope. Panels **A**, **B**, **C,** and **D**: are phase images of Y79 cells unstained, treated with free Dox, Scr-EpDT3-Dox or Scr-EpDT3-Dox, and the respective right panel to it is fluorescent image merged.

EpDT3-Dox showed the target-specific binding and delivery of Dox in vitro. Microscopic images with free Dox-treated cells clearly show Dox localization in the nucleus at 2 h for the Müller glial cells and the Y79 cells (Figure 6B and Figure 7B), whereas with EpDT3-Dox, the localization was observed in the cytoplasm, faintly in the nucleus of the Y79 cells at 2 h (Figure 7C), and no such staining pattern was observed for the Müller glial cells (Figure 6D,H). The Scr-EpDT3-Dox conjugate showed marginal or no binding on the Müller glial cells and the Y79 cells (Figure 6C and Figure 7D). After the cells were incubated for 12 h post treatment with the aptamer-Dox conjugates, localization for cells treated with EpDT3-Dox was mainly on the nucleus in the Y79 cells whereas no staining was observed in the Müller glial cells (Figure 6H and Figure 8C). However, Scr-EpDT3-Dox did not show any detectable binding on either cell line (Figure 6G and Figure 8D).

### Effect of aptamer-doxorubicin conjugate on cell cytotoxicity

**Figure 9:**
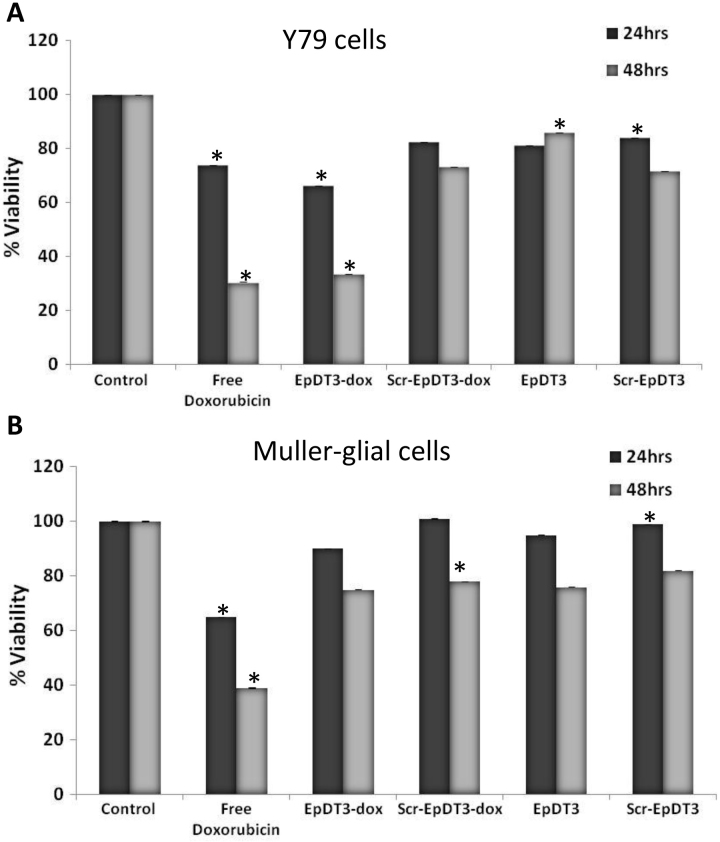
Cell proliferation inhibition by aptamer-Dox conjugates in vitro. The Y79 and Müller glial cells were seeded 24 h before treatment with free Dox, and aptamers (EpDT3 and Scr-EpDT3) and aptamer-Dox conjugates (EpDT3-Dox and Scr-EpDT3-Dox; n=3) were added to cells in serum-free condition for 2 h followed by media change with 10% FBS. Cells were further incubated for 24 h and 48 h, and the MTT assay was performed. **A**: The graph represents the percent viable Y79 cells after 24 h and 48 h of treatment. Cytotoxicity was observed with cells treated with free Dox and EpDT3-Dox. **B**: Graph showing percentage viability of Müller glial cells treated with aptamer and aptamer-Dox. Only free Dox treated cells exhibit significant cytotoxicity.

**Figure 10:**
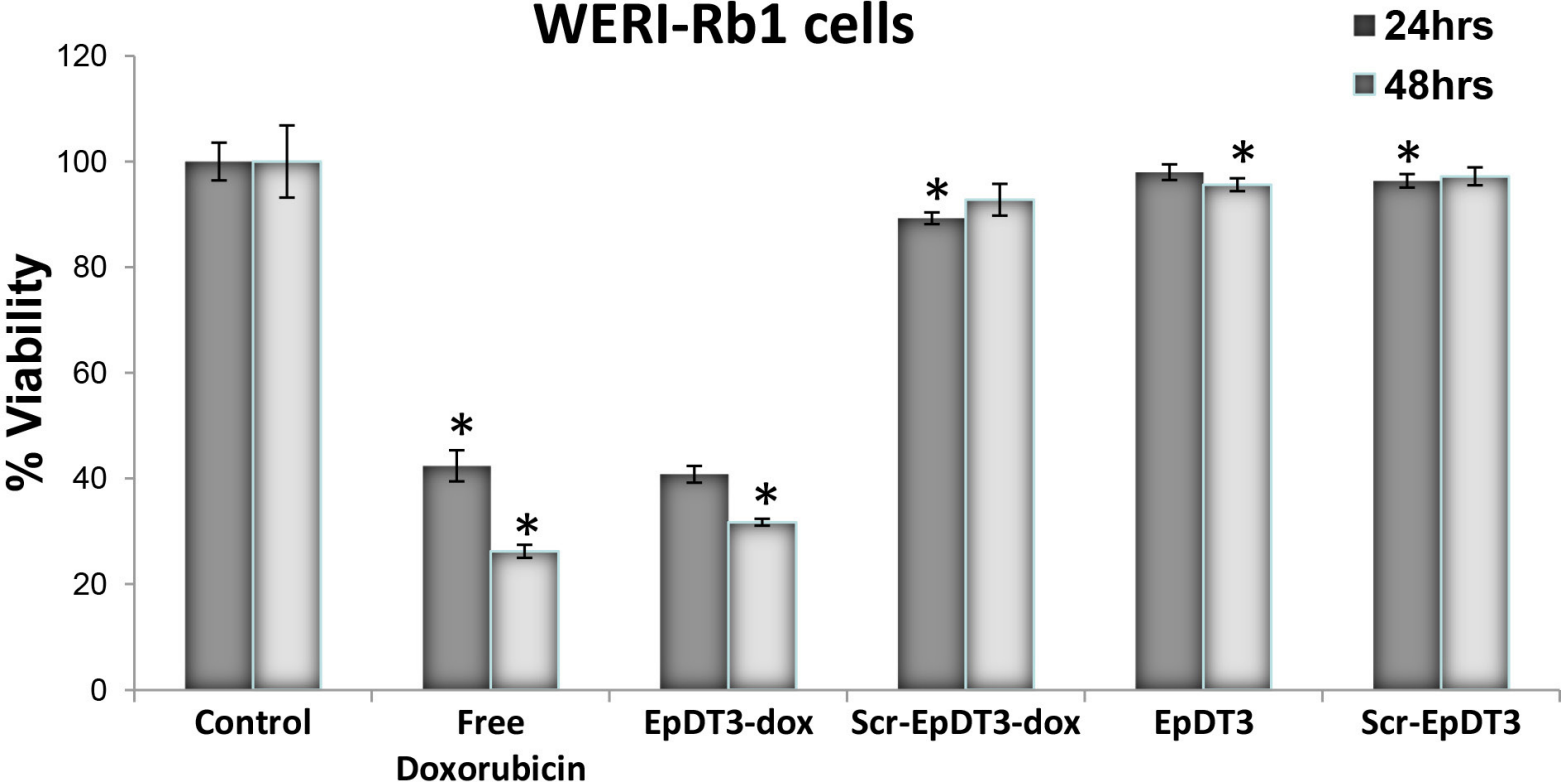
Cell proliferation inhibition by aptamer-Dox conjugates in vitro. WERI-Rb1 cells were seeded 24 h before treatment with free Dox, and aptamers (EpDT3 and Scr-EpDT3) and aptamer-Dox conjugates (EpDT3-Dox and Scr-EpDT3-Dox; n=3) were added to cells in serum-free condition for 2 h followed by media change with 10% FBS. Cells were further incubated for 24 h and 48 h, and an MTT assay was performed. The graph represents the percent viable WERI-Rb1 cells after 24 h and 48 h of treatment. Cytotoxicity was observed with cells treated with free Dox and EpDT3-Dox.

Cell cytotoxicity was evaluated by monitoring the metabolic rate of the cells with an MTT assay. Free Dox showed toxicity in the cancerous and normal cell lines (Figure 9). Free Dox showed 27% and 35% cytotoxicity at 24 h and 70% and 60% cytotoxicity at 48 h post treatment on the Y79 and Müller glial cells, respectively. The EpDT3-Dox conjugate showed higher cytotoxicity in the cancerous Y79 cell line compared to the noncancerous Müller glial cells. The non-chimeric aptamer alone exhibited reduced cellular toxicity compared to the aptamer alone. The EpDT3-Dox conjugate showed 33% and 10% cytotoxicity at 24 h and 66% and 25% cytotoxicity at 48 h on the Y79 and Müller glial cells, respectively. The EpDT3-treated cells showed 19% and 5% cytotoxicity at 24 h and 14% and 24% cytotoxicity at 48 h post treatment on the Y79 and Müller glial cells, respectively. The Scr-EpDT3-Dox conjugate and Scr-EpDT3 showed 18% and 16% cytotoxicity and 27% and 28% cytotoxicity at 24 h and 48 h on the Y79 cells. No cytotoxicity was observed at 24 h while 22% and 18% cytotoxicity was observed at 48 h on the Müller glial cells (Figure 9A,B). Free doxorubicin showed 57% and 73% cytotoxicity toward the WERI-Rb1 cells at 24 h and 48 h, respectively. EpDT3-Dox and Scr-EpDT3-Dox showed 59% and 68% cytotoxicity and 96% and 97% cytotoxicity on the WERI-Rb1 cells, respectively (Figure 10).

## Discussion

EpCAM is a putative stem cell marker in breast, liver, colon, pancreas, and prostate tumors [24,40,41]. Recently, our group showed the correlation and presence of EpCAM and coexpression among the CSC markers [32]. EpCAM+ breast cancer and hepatocellular carcinoma showed the CSCs or CPCs phenotype [19,20]. Hence, we used the EpCAM-targeted therapeutic approach for retinoblastoma using an aptamer against EpCAM, and this is the first study using the EpCAM aptamer for targeted drug delivery in RB cells. EpCAM is ideal for drug targeting in RB because as this molecule is overexpressed in invasive tumors and is a putative cancer stem cell marker. The results clearly show a significant amount of EpCAM antigen was present in the Y79 and WERI-Rb1 cell lines compared to the Müller glial cells (Figure 1). In addition, the binding potential of EpDT3 and Scr-EpDT3 checked against RB fresh tumors, Y79 and WERI-Rb1, RB cells and Müller glial cells, showed 35% positive population in the retinoblastoma tumor cells (n=4) and the RB cell lines (Figure 2 and Figure 3). This might be due to the heterogeneous population of cells in the tumor and cell lines expressing EpCAM. This is consistent with our previous observation that EpCAM is expressed only in a subset of population of RB cell lines and only EpCAM^+^ Y79 cells have properties of CSCs [32].

The EpCAM protein is overexpressed in RB cell lines. EpDT3-FI showed binding only to the RB cells and not to the Müller glial cells, indicating the cancer cell–specific expression of EpCAM. In contrast, no binding was observed for the scrambled aptamer in the primary RB cells, Y79 and WERI-Rb1, and the Müller glial cells (Figure 2 and Figure 3). This is in agreement with previous observations that 2’-OMethyl modification of the pyrimidines in an aptamer hampers binding of the aptamer to the EpCAM receptor [35].

The optimal performance of the equimolar Dox and aptamer agrees with theoretical prediction of one Dox site in the aptamer (Figure 4A). The PSMA aptamer for Dox delivery had a single site predicted theoretically for the Dox conjugation [38,42]. However, the Dox-to-aptamer ratios varied (0.5–2) in different practical applications. The slow diffusion of Dox from the aptamer-Dox conjugates compared to the free Dox is attributed to the physically bound state of Dox to the aptamer (Figure 6). Similar results were observed by Banglok et al. [42]. The free Dox localized to the nucleus in the RB and Müller glial cell lines. The nucleocytoplasmic presence of Dox in the Y79 cells and not in the Müller glial cells incubated with EpDT3-Dox. This indicates that the conjugation of the EpDT3 aptamer to the Dox did not impair the target finding ability of the Dox. The inability of Scr-EpDT3-Dox to localize to the nucleus indicates the targeted binding of the EpDT3 aptamer over the control aptamer. The target-specific binding of EpDT3 to EpCAM, a membrane antigen, resulted in the internalization of the aptamer-drug conjugate into the cytoplasm and finally into the nucleus resulting in sustained drug delivery to the nucleus of cells expressing EpCAM (Figure 6, Figure 7, and Figure 8). Other studies have obtained similar results in LNCaP and CCRF-CEM cancer cell lines [12,38]. EpDT3-Dox and Scr- EpDT3-Dox did not bind or get internalized in the Müller glial cells, proving the selective binding of the aptamer to the cancerous cells sparing the normal cells.

The efficacy of the EpDT3-Dox drug delivery system in killing the Y79 cells and the WERI-Rb1 cells, and not the noncancerous Müller glial cells (Figure 9A,B and Figure 10) indicates the cancer cell–specific targeting of the drug. The aptamer binding to Dox spared the drug delivery to the normal cells and killed the cancer cells precisely. Therefore, EpDT3-Dox may reduce undesirable side effects associated with chemotherapy. The Scr-EpDT3-Dox conjugate and the aptamer alone did not have a marked effect in inhibiting cell proliferation indicating the specificity of EpDT3 binding to the EpCAM-positive cells alone.

In conclusion, we have engineered a chimeric aptamer that binds to its target molecule and efficiently delivers the drug to the cancer cells. The aptamer-based targeted drug delivery prevents off-target effects of the drug Dox. This Dox conjugate can be applied as a therapeutic agent in all cancers overexpressing EpCAM. EpCAM aptamer–based drug delivery in the future can be potentially exploited with stable linking of the drugs for targeting EpCAM-positive cancer stem cells in RB as well as in other cancers. The aptamer-conjugated nanocarriers can be used for imaging tumors or as therapeutic systems for targeting EpCAM using chimeric aptamer-small interfering RNA for RB.
